# Nontarget Analysis of the Volatile and Semivolatile Chemical Profile of Wildland Firefighter Gear: Insight into Occupational Exposure and Health Implications

**DOI:** 10.1021/acs.est.6c10862

**Published:** 2026-08-21

**Authors:** Patrica-Ivy Agorsor, Husam Kafeenah, Margaret D. Taiwo, Robin Verble, Michael O. Eze

**Affiliations:** † Department of Chemistry, 14717 Missouri University of Science and Technology, Rolla, Missouri 65409, United States; ‡ Department of Biological Sciences, Missouri University of Science and Technology, Rolla, Missouri 65409, United States; § Center for Research in Energy and Environment, Missouri University of Science and Technology, Rolla, Missouri 65409, United States; ∥ Center for Biomedical Research, Missouri University of Science and Technology, Rolla, Missouri 65409, United States

**Keywords:** prescribed fire, personal protective equipment (PPE), occupational exposure, secondary exposure, toxicants, decontamination, midwestern U.S

## Abstract

Prescribed fire releases a complex mixture of volatile and semivolatile organic compounds to the environment. Firefighters are directly exposed to these hazardous mixtures through occupational activities. This study investigated the volatile and semivolatile chemical contamination profile of firefighter protective gear and the effectiveness of laundering as a decontamination strategy. Our results revealed that firefighter gear accumulated a broad spectrum of combustion-derived contaminants, predominantly composed of polycyclic aromatic hydrocarbons and derivatives, phenolic compounds, and aromatic hydrocarbons. Representative compounds identified included toluene, biphenyl, naphthalene, 1-methynaphthalene, 2-methylnaphthalene, acenaphthene, phenol, alkylated phenols, and chlorinated phenols. Quantitative analysis revealed representative combustion-derived compounds at concentrations (mean ± standard deviation, SD) including toluene (0.958 ± 0.293 ng/g), biphenyl (0.015 ± 0.004 ng/g), naphthalene (0.053 ± 0.020 ng/g), 1-methynaphthalene (0.468 ± 0.132 ng/g), 2-methylnaphthalene (0.009 ± 0.002 ng/g), and acenaphthene (0.303 ± 0.001 ng/g). Multivariate analysis showed that although laundering substantially reduced contaminant levels, residues of several compounds remained significantly detectable in washed samples. These results demonstrate that firefighter personal protective equipment (PPE) serves as a reservoir for toxic compounds, underscoring the additional risk of secondary exposure to firefighters through dermal contact, handling, and off-gassing. Together, our findings emphasize the need for the safe transport of protective gear (e.g., in sealed bags), proper storage before laundering, and periodic replacement of these protective materials. This will help mitigate secondary exposure and adverse long-term health effects.

## Graphical abstract

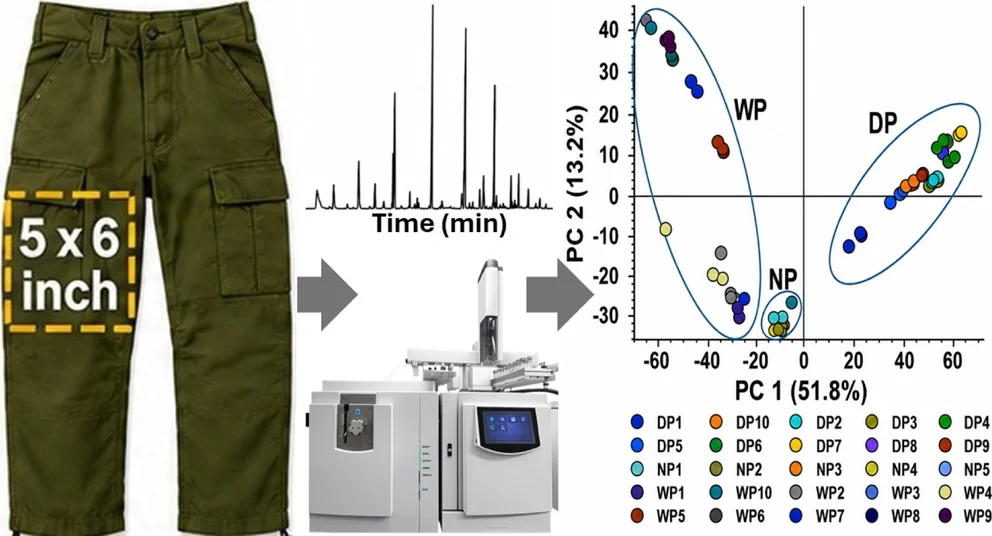

## 1 Introduction

Prescribed fire, also known as controlled burning, is the intentional application of fire conducted when weather and environmental conditions meet specified burn plan criteria to achieve specific land management objectives. It is widely used in forestry, agriculture, and rangeland management to reduce hazardous fuel loads, maintain ecosystem health, promote biodiversity, and recycle nutrients into the soil.^1^ Unlike wildfires, prescribed burns are conducted when weather conditions, fuel characteristics, and topographic features fall within an established burn plan, with fire crews monitoring ignition and spread to minimize risk. Despite these precautions, prescribed burns release smoke that contains a complex mixture of particulate matter and organic compounds, many of which are potentially hazardous to human health.^2^ Compounds produced during prescribed burning are formed primarily through the thermal decomposition and incomplete combustion of cellulose, hemicellulose, and lignin. In addition, both the local position on a wildland fire and the broader geographic context influence smoke exposure. Polycyclic aromatic hydrocarbons (PAHs) are a major class of aromatic compounds generated by incomplete combustion. Many PAHs are mutagenic and genotoxic because they are metabolized into reactive intermediates that form DNA adducts. Several of these are classified as carcinogenic in animals and are considered possible or probable human carcinogens.^3,4^ While a previous study of prescribed fires in the midwestern United States focused on particulate matter (PM), carbon monoxide, and black carbon,^5^ little is known about the organic compound profile of prescribed fires in the midwestern U.S. states. In comparison to wildfires, prescribed fires were found to have significantly higher concentrations of PM_2.5_ than wildfire smoke.^6–9^ Thus, studies of prescribed burning are an important context for studying occupational and environmental health risks of airborne organic pollutants.

Prescribed burning may be particularly hazardous to wildland firefighters. Wildland firefighters are directly exposed to these complex mixtures of volatile and semivolatile organic compounds during prescribed burns, with contaminants spanning diverse chemical classes and mechanisms of toxicity.^10,11^ These substances can be deposited onto and absorbed into personal protective equipment (PPE), particularly fire-resistant pants and shirts, which are designed to provide thermal and physical protection. While PPE offers critical protection against heat and may provide limited protection against particulates, repeated use can result in the accumulation of chemical residues on firefighter gear, as previously reported in structural firefighting studies, although contamination patterns in wildland prescribed burning remain less characterized.^12^ Wildland firefighter pants may be even more permeable to chemicals due to their single-layer design. Such contaminants may transfer to the skin during wear or off-gas during storage, prolonging exposure even outside of active firefighting contexts.^10^ Notably, several epidemiological studies have reported elevated cancer risks among firefighters, with occupational exposures suspected to play a significant role.^13^ In 2022, the International Agency for Research on Cancer (IARC) classified occupational exposure as a firefighter as carcinogenic to humans (group 1) based on sufficient evidence for mesothelioma and bladder cancer and limited evidence for several additional cancers.^14^ To mitigate exposure, decontamination practices such as laundering are commonly implemented. However, their effectiveness varies by chemical properties, fabric composition, and cleaning protocol. Studies have shown that some combustion-derived contaminants, including PAHs and other semivolatile organic compounds, are only partially removed following laundering activities.^15,16^

Characterizing PPE contamination patterns, particularly the differences between new, used, and washed gear, is essential for evaluating firefighter exposure risks and improving decontamination strategies. In this study, using high-resolution mass spectrometry, we conducted a nontarget analysis of the volatile and semivolatile organic compounds associated with fabric patches attached to wildland firefighter protective pants before exposure, after wildfire exposure, and following laundering to gain insight into contamination variability, identify chemical groups associated with prescribed burning, and evaluate laundering effectiveness.

## 2 Materials and Methods

### 2.1 Chemicals and Reagents

Methanol (MeOH) and acetonitrile (ACN) (optima LC/MS grade, ≥99.9% purity, Fisher Chemical) were used as extraction solvents. Sodium chloride (NaCl) (≥99.5% purity) and deuterated internal standards (toluene-*d*8, naphthalene-*d*8, and acenaphthene-*d*10) were purchased from Sigma-Aldrich (St. Louis, MO, USA). Ultrapure water was obtained from a Milli-*Q* system. Solid-phase microextraction (SPME) fiber (80 μm DVB/carbon WR/PDMS) was purchased from Thermo Fisher Scientific (Waltham, MA, USA). Headspace screw cap vials (20 mL, 23 × 75 mm, clear glass, PFTE/silicone septa) were acquired from Supelco (Millipore Sigma, Bellefonte, PA, USA).

### 2.2 Fuel Composition, Firefighter Gear and Exposure Characteristics

In this study, new (NP), dirty (DP) (worn during operation), and washed (WP) wildland firefighter pant fabric samples were evaluated. The pants were composed of 93% Nomex IIA (a fabric composed of meta-aramid fibers made from poly­(*m*-phenylene isophthalamide)), 5% Kevlar IIIA, and 2% antistatic fibers designed to resist melting, dripping, and burning as set forth in the standards for federal wildland firefighter protective gear (NFPA 1977). To minimize variation associated with the individual firefighter activities and exposure conditions, all fabric samples were exposed during the same prescribed fire operation. The operation consisted of prescribed burns conducted in an upland oak-hickory forest in April 2025. Two categories of wildland firefighter pants were evaluated: noncolored (olive green) and colored (forest green). A total of 17 replacement fabric patches (10 noncolored and 7 colored) were deployed and worn by 17 different firefighters during the prescribed burn. Prior to the burn, one original front pocket section from each firefighter’s pants was removed and replaced with a newly prepared fabric patch of matching material and dimensions (5 × 6 in.). Each replacement patch represented an independent exposure sample and remained on an individual firefighter’s pants throughout the prescribed burn. Five additional new, unexposed fabric patches of each material type were retained as controls (NP). During the prescribed burn, firefighters performed their routine assigned operational duties at multiple locations across the burn unit but did not participate in ignition activities, thereby minimizing direct contamination from torch fuel exposure. Following exposure, each exposed patch was subsequently sealed in Ziplock bags to minimize cross-contamination during transport and storage. Each exposed patch was then divided into two equal portions (5 × 3 in.), with one portion analyzed as the dirty-pants (DP) sample and the second portion subjected to laundering prior to analysis as the corresponding washed-pants (WP) sample. Fabric patches were destructively sampled for solvent extraction and chemical analysis. On washing, DP1-DP10 became WP1-WP10, and DP11–17 became WP11-WP17. Washed pants were washed in warm water with a commercial detergent. All samples were washed singly in a Kenmore automatic machine washer. The wash cycle lasted approximately 50 min and was conducted using warm/hot water temperature (∼60 °C) consistent with recommended laundering practices for Nomex firefighter protective fabrics. The detergent employed was Tide Free and Clear, a commercial surfactant formulation designed for low-allergen, fragrance-free cleaning. Samples were machine-dried on a dryness-sensing automatic setting at normal heat levels prior to extraction and chemical analysis.

### 2.3 Sample Extraction

Fabric samples were extracted using a 1:1 mixture of methanol and acetonitrile (20 mL MeOH and 20 mL ACN; 40 mL total extraction volume). Samples were immersed in the extraction solvent and sonicated for 1 h to effectively extract contaminants from the fabric. To assess background interactions and baseline signals, solvent matrix blanks consisting of extraction solvents and Milli-*Q* water were prepared alongside the samples. Following sonication, samples were vortexed for 1 min to ensure thorough mixing. The fabric material was then removed from the extraction solution, and the final recovered extract volume after extraction was approximately 34–35 mL. Before headspace analysis, 200 μL aliquot of the extract was transferred into a 20 mL headspace vial containing 4.8 mL Milli-*Q* water and 2.0 g of sodium chloride was added to induce salting out, which helps promote analyte partitioning into the headspace. Volatile organic compounds were extracted using the headspace solid-phase microextraction (HS-SPME) technique. Samples were incubated at 65 °C for 10 min under continuous agitation, and analytes were extracted using a preconditioned 80 μm DVB/Carbon WR/PDMS fiber prior to GC–MS analysis (Figure 1).

**Figure 1:**
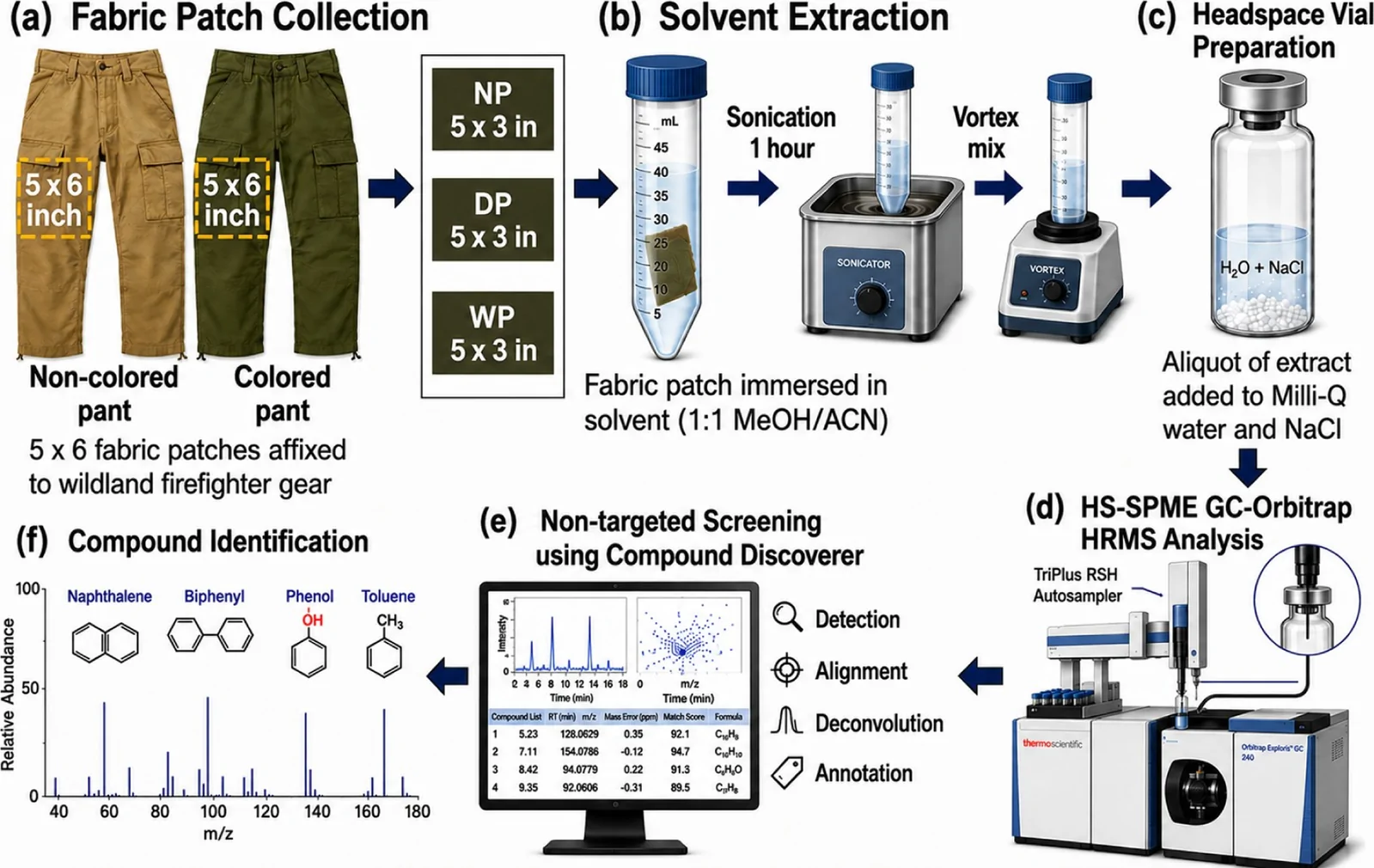
Workflow for fabric sample collection, extraction, and nontargeted chemical analysis of wildland firefighter protective pants. (a) Fabric patches were collected from new (NP), dirty/exposed (DP), and washed (WP) noncolored and colored firefighter pants. (b) Fabric samples were extracted using methanol/acetonitrile (1:1, MeOH/ACN), followed by sonication and vortex mixing. (c) An aliquot of the extract was transferred to a headspace vial containing Milli-*Q* water and NaCl. (d) Volatile and semivolatile organic compounds were analyzed using headspace solid-phase microextraction coupled with GC–orbitrap high-resolution mass spectrometry (HS-SPME GC–orbitrap HRMS). (e) Nontargeted data processing was performed using compound discoverer for feature detection, alignment, deconvolution, and annotation. (f) Compounds were identified using high-resolution mass spectral information and spectral library matching.

### 2.4 Semiquantitative Analysis

To evaluate the accumulation and removal of selected combustion-derived contaminants on firefighter textile samples, a semiquantitative analysis was performed using deuterated internal standards (toluene-*d*8, naphthalene-*d*8, and acenaphthene-*d*10). Each internal standard was added to the headspace vial containing the sample extract at a known concentration prior to HS-SPME GC–MS analysis. Semiquantitative concentrations were determined using the ratio of the peak area of the target analyte to that of the deuterated internal standard. Concentrations were normalized to the original fabric mass and expressed as nanograms per gram (ng/g) of textile. We ensured that the operational signal was within the instrument’s limits and that analyte peaks were clearly visible above the background baseline noise (at least a 3:1 signal-to-noise ratio). Because analyte-specific calibration curves, linear dynamic ranges, and method detection limits were not established, the reported values represent semiquantitative concentrations. These estimates were used to compare relative contaminant accumulation and removal among NP, DP, and WP sample groups rather than absolute concentrations.

### 2.5 GC–MS and Data Analysis

Gas chromatography–mass spectrometry (GC–MS) analysis was performed using a Trace 1610 GC coupled to an Orbitrap Exploris MS (Thermo Fisher Scientific, Waltham, MA, USA) equipped with a TriPlus RSH SMART automatic sampler with headspace injection capability. Chromatographic separations were carried out on a TG-5SilMS column (30 m × 0.25 mm inner diameter, 0.25 μm film thickness) (Thermo Fisher Scientific). Analyses were conducted using electron ionization (EI) in positive polarity mode at an electron energy of 70 eV. Splitless injection was employed during EI analysis. Helium (99.999% ultrapure purity) was used as the carrier gas, while nitrogen gas (99.999% ultrapure purity) was used for the C-trap cell. The injection port temperature was 225 °C. The carrier gas flow rate was held constant at 1.2 mL/min, with a front inlet purge flow of 5.0 mL/min. The initial column temperature was 40 °C for 0.5 min, then ramped to 280 °C at a rate of 25 °C/min, followed by a final hold for 6 min. The MS transfer line and ion source temperatures were both maintained at 280 °C. Mass spectrometric data were acquired in full-scan mode with an *m*/*z* range of 50–500, with mass resolution set at 30,000 fwhm (full width at half-maximum). The total ion chromatogram (TIC) minimum intensity threshold was set at 1 × 10^6^, and the mass tolerance window was maintained at ± 5 ppm. Retention indices (RI) of unknown compounds were determined using an *n*-alkane standard series (C_8_–C_20_) analyzed under identical chromatographic conditions. Chromeleon software (Thermo Fisher Scientific) was used for instrument control and data acquisition. Raw data files were processed using Compound Discoverer 3.3.3.2 (Thermo Fisher Scientific) for peak extraction, alignment, deconvolution, identification, and annotation. GC-EI deconvolution and compound annotation were performed using optimized peak detection, grouping, and high-resolution spectral matching parameters summarized in Table S1. Compound identification was achieved through spectral matching against the NIST main library and GC-Orbitrap contaminants library databases using Compound Discoverer 3.3.3.2. Selected compounds were further confirmed and quantified using deuterated internal standards. Statistical analysis and data visualization were performed using R software and Compound Discoverer 3.3.3.2.

### 2.6 Compound Identification and Annotation

A nontargeted GC-EI Orbitrap workflow was employed to characterize volatile and semivolatile organic compounds associated with wildfire exposure on firefighter protective textiles, similar to previous high-resolution GC–MS nontarget screening workflows.^17^ Compound annotation was performed using Compound Discoverer 3.3.3.2 in combination with the NIST Main Library and GC-Orbitrap Contaminants Library databases, consistent with previous high-resolution GC–MS nontarget screening workflows. Identification confidence was evaluated using complementary criteria, including accurate mass agreement, calculated and NIST-observed molecular weights, retention index (RI) agreement, search index (SI), high-resolution filtering (HRF), and total annotation scores.^18,19^ Compounds with annotation scores ≥70, SI and RSI values ≥700, and HRF scores ≥70 were considered putatively identified. Representative examples of the annotation workflow and deconvoluted EI spectra are presented in Figure 2 for naphthalene, 1-methylnaphthalene, and 2-methylnaphthalene. These compounds exhibited strong spectral matching parameters, including SI values ranging from approximately 955–960, and HRF scores greater than 95, indicating high-confidence annotation. The deconvoluted EI spectra demonstrated characteristic molecular and fragment ions consistent with aromatic hydrocarbon fragmentation patterns reported in the NIST spectral database. For example, naphthalene exhibited a characteristic molecular ion at *m*/*z* 128.0618 corresponding to C_10_H_8_, while 1-methylnaphthalene and 2-methylnaphthalene showed molecular ions at *m*/*z* 142.0777 and 142.0778, respectively, consistent with C_11_H_10_ aromatic structures. Agreement between experimental and library retention indices further strengthened compound assignment reliability. The combined use of high-resolution accurate mass measurements, spectral matching metrics, deconvolution analysis, retention index confirmation, and optimized signal-processing parameters improved confidence in compound identification.

**Figure 2:**
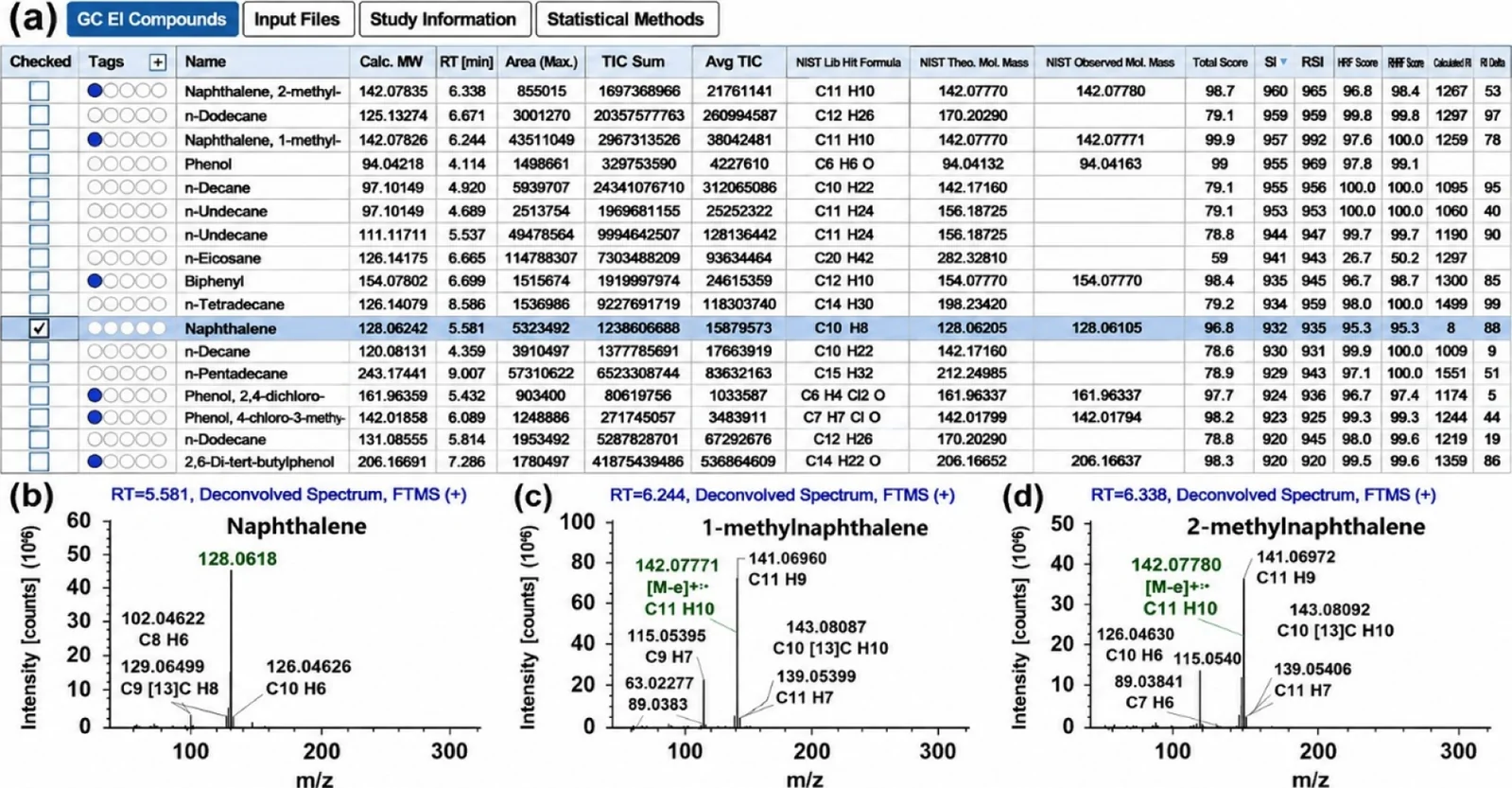
Representative (a) compound annotation workflow and deconvoluted EI spectra for (b) naphthalene, (c) 1-methylnaphthalene, and (d) 2-methylnaphthalene demonstrating characteristic fragmentation patterns and high-confidence spectral agreement with NIST library reference spectra.

## 3 Results and Discussion

### 3.1 Chemical Profiles of Firefighter Gear

A comparative analysis of wildland firefighter pants revealed distinct exogenous chemical profiles based on exposure. The overall chemical profile was strongly influenced by exposure condition, with new pants (NP), dirty pants (DP), and washed pants (WP) exhibiting distinct clustering patterns. Principal component analysis (PCA) score plots showed a clear separation between DP, WP, and NP samples for both noncolored and colored firefighter pants (Figure 3), indicating that occupational exposure during wildfire suppression substantially altered the chemical composition of firefighter gear. The separation of DP from NP provides evidence of occupationally derived contamination, while the shift of WP toward NP indicates a reduction in chemical burden following laundering. The first principal component (PC1) accounted for 51.8% and 42.2% variance for noncolored and colored pants, respectively, indicating a strong separation of DP from WP, while also reflecting variability in contaminant accumulation and retention among samples.

**Figure 3:**
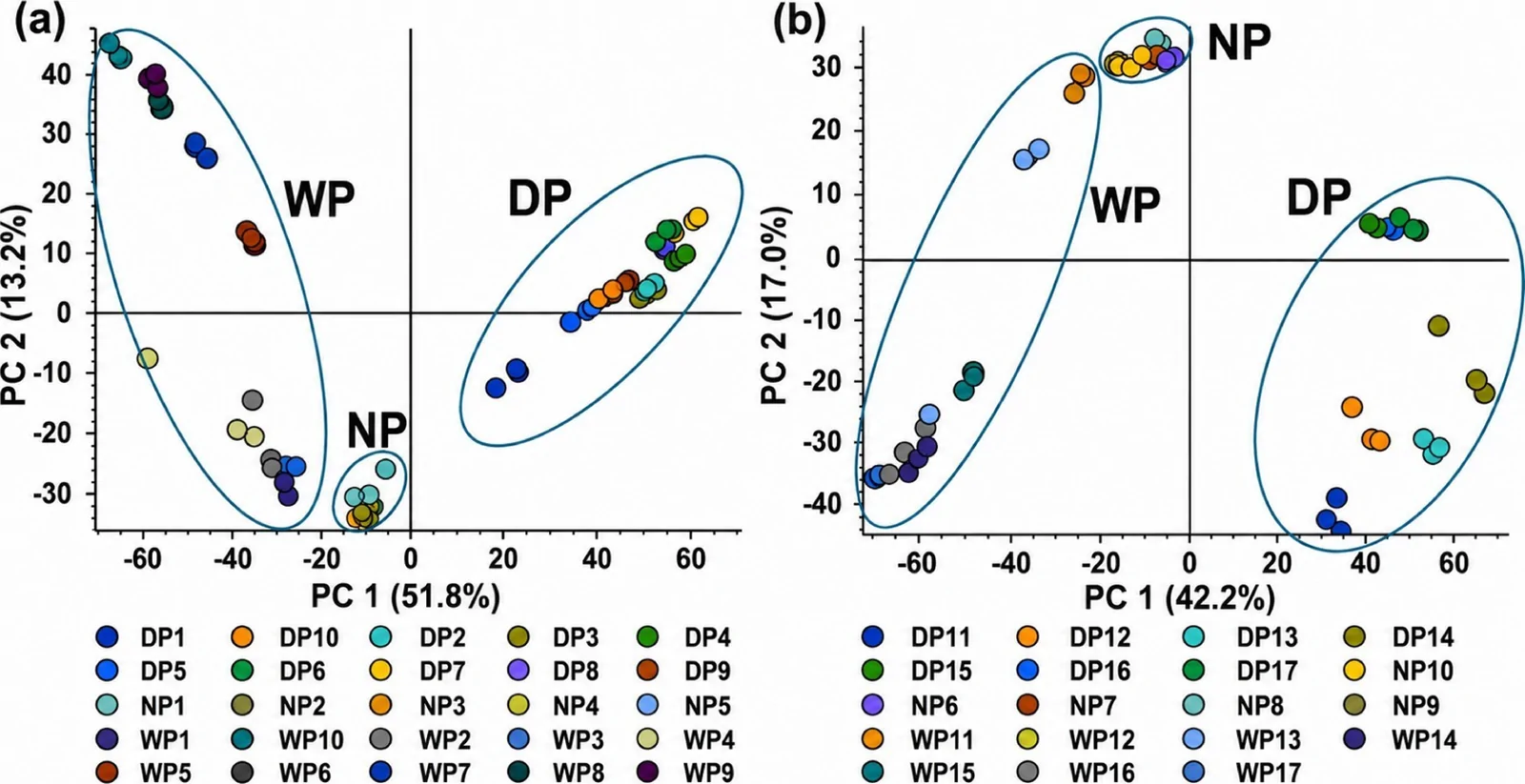
Principal component analysis of chemical profiles of (a) noncolored and (b) colored firefighter protective pants grouped by exposure condition. DP: dirty pants; NP: new pants; WP: washed pants.

Across all PCA comparisons, the dominant factor driving chemical differences was the condition of the pants (dirty versus washed), rather than sampling location or replicate set. Laundering significantly modified these profiles but did not fully eliminate all compounds. Since newly attached fabric patches matching the original pocket dimensions replaced the existing firefighter pants pockets before exposed to the same prescribed burns having the same fuel composition, the dispersion observed among dirty pants (noncolored vs colored) reflects heterogeneity in fireground exposure and contaminant deposition. Consistent with this, colored pants showed greater chemical diversity and lower variance along PC1 compared to noncolored pants, reflecting differences in contaminant retention behavior potentially associated with fabric composition, dye chemistry, or surface characteristics. Although prescribed burn conditions were generally similar across sampling events, contaminant deposition onto firefighter clothing may still vary due to differences in direct exposure to soot, aerosols, and condensable vapors at the fireground.^10,20^ Similar variability in firefighter exposure profiles has been reported in previous wildland firefighter studies, where contaminant accumulation differed according to task assignment, proximity to active combustion, wind direction, smoke intensity, and duration of exposure.^21–23^ Other factors, such as sweat, skin contact, and secondary transfer mechanisms, may contribute to variability in chemical profiles.

Hierarchical clustering heat maps revealed that the new pants (NP) exhibited distinct clustering compared to the soiled pants (DP) reflecting their relatively low compound abundance, as indicated by the predominance of green coloration across (Figure 4a). Dirty pants showed elevated levels of several compounds (red zones), which clustered away from NP and WP samples, suggesting significant accumulation of chemical residues during firefighting activities. The more pronounced chemical intensity of DP (in Figure S1a) is in harmony with its greater variability observed in the PCA plots (in Figure 3), reflecting cumulative burden. WP samples generally exhibited lower chemical intensities than DP samples, confirming that laundering substantially reduced the contaminant burden. However, several WP samples, particularly among the colored pants (Figure S1a), retained detectable chemical signals, suggesting the persistence of residual contaminants and possible localized influences associated with detergent residues or incomplete removal of strongly retained compounds. The analysis of the chemical compositions of the noncolored and colored pants revealed common chemical fingerprints consistent with PAH combustion byproducts and other fire-related contaminants (Figure 4b).

**Figure 4:**
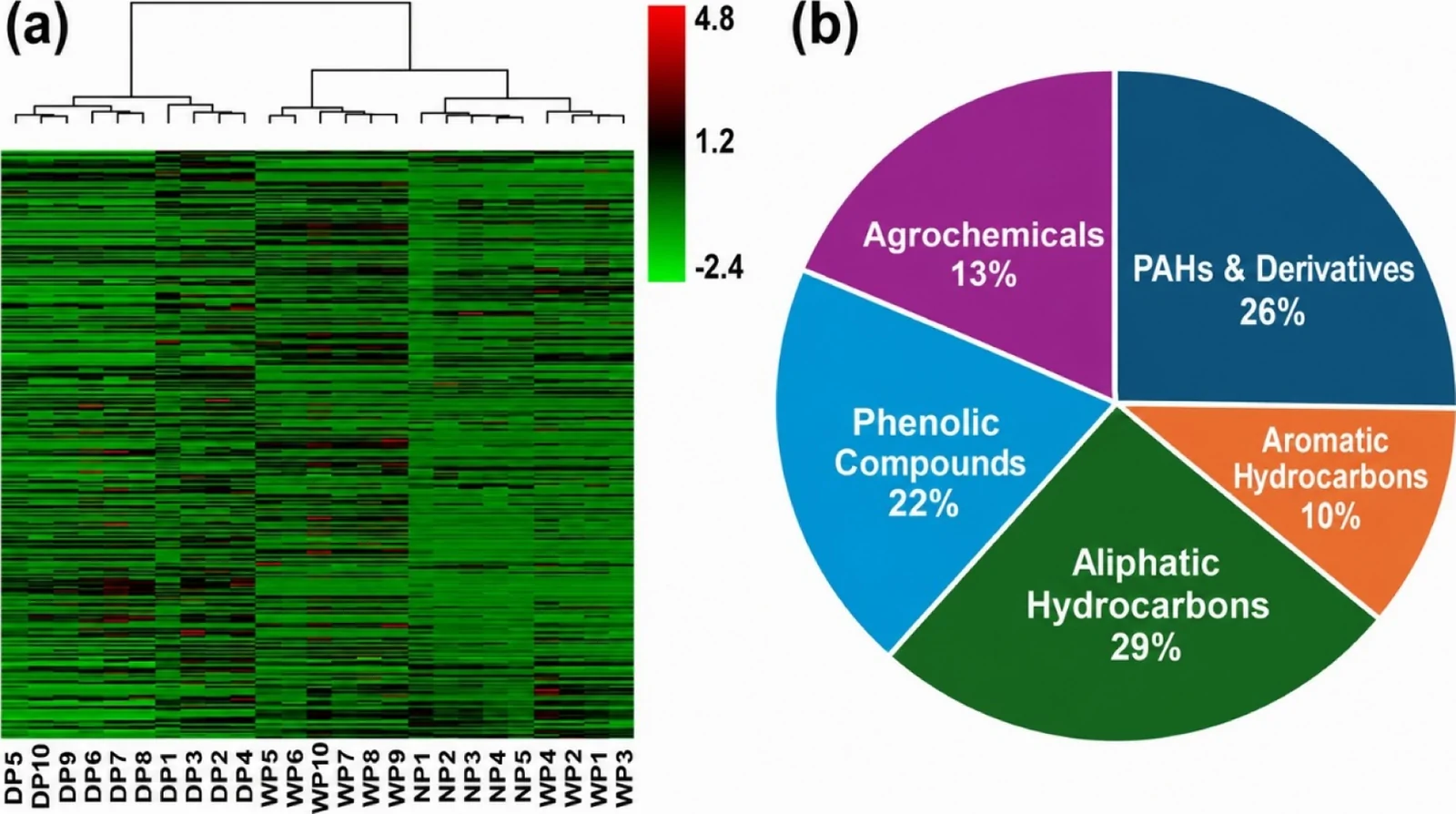
Chemical profile of prescribed fire. (a) Heat map showing hierarchical clustering of new, washed, and dirty noncolored pants. The color scale indicates relative chemical abundance, with red signifying high chemical intensity. (b) Percentage distribution of the dominant chemical class in the noncolored firefighter pants. NP: new pants; WP: washed pants; DP: dirty pants.

Our results revealed a clear dominance of aliphatic hydrocarbons (29%) in the chemical profile of firefighter gear. Polycyclic aromatic hydrocarbons (PAHs) and derivatives accounted for 26% of all identified contaminants, followed by phenolic compounds (22%), aromatic hydrocarbons (10%), and the remaining agrochemical-related compounds in noncolored pants (Figure 4b). This is consistent with the chemistry of organic matter combustion.^10,11^ Aliphatic hydrocarbons, especially *n*-alkanes, can arise from thermal degradation of plant waxes and other natural organic matter. Due to their semivolatile nature, these compounds can partition onto particulate matter and subsequently deposit onto materials. PAHs and related derivatives included fluorene, acenaphthylene, naphthalene, 1-methylnaphthalene, 2-methylnaphthalene, acenaphthene, and phenanthrene (Table 1). PAHs and alkylated PAHs are characteristic products of incomplete combustion, and their occurrence on firefighter gear agrees with reports of PAH exposure among wildland firefighters and prescribed-burn personnel^21,23–25^ Aside from aliphatic hydrocarbons and PAHs, notable amounts of phenolic compounds and aromatic hydrocarbons, including BTEX compounds and alkyl-substituted BTEX compounds were also detected. These include biphenyl, toluene, (1,1-dimethylpropyl)­benzene, and pentylbenzene (Table 1). The presence of BTEX and alkylated BTEX compounds is consistent with the volatilization and sorption of light aromatic hydrocarbons, which readily partition to fabrics due to their high vapor pressures and abundance in smoke plumes.^26^

**Table 1:** Key Compounds in Firefighter Pants, Their Classification, and Toxicity Profile^a^

S/N	compound name	molecular formula	molecular weight	RT (min)	annotation score	SI	pollutant group	toxicity profile
1	toluene	C_7_H_8_	92.0618	2.724	76.5	808	BTEX	respiratory and skin irritant. CNS depressant (headache, dizziness). neurobehavioral effects. Liver and kidney toxicant
2	pentylbenzene	C_11_H_16_	148.1258	5.536	77.4	849	alkyl BTEX	eye, skin, and respiratory tract irritant
3	biphenyl	C_12_H_10_	154.0780	6.699	98.4	935	PAH	eye, skin, and respiratory tract irritant. liver, kidney, and CNS toxicant
4	baphthalene	C_10_H_8_	128.0624	5.581	96.8	932	PAH	group 2B carcinogen. causes respiratory, hematological, and neurological effects
5	1-methyl-naphthalene	C_11_H_10_	142.0783	6.244	99.9	957	PAH	respiratory irritant. causes neurological defects
6	2-methyl-naphthalene	C_11_H_10_	142.0784	6.338	98.7	960	PAH	liver and kidney toxicant. CNS depressant
7	acenaphthylene	C_12_H_8_	152.0625	7.968	92.8	702	PAH	eye, skin, and respiratory tract irritant
8	acenaphthene	C_12_H_10_	154.0781	8.590	98.8	722	PAH	eye, skin, and respiratory tract irritant
9	fluorene	C_13_H_10_	166.0783	9.126	91.2	729	PAH	eye, skin, and respiratory tract irritant. Respiratory effects include pulmonary edema
10	phenanthrene	C_14_H_10_	178.0791	10.251	84.5	768	PAH	respiratory irritant. Generally low acute and chronic toxicity
11	phenol	C_6_H_6_O	94.0416	4.105	99.6	972	phenolics	skin irritant. Liver, kidney, and CNS toxicant
12	3-methyl-phenol	C_7_H_8_O	108.0570	5.180	95.6	830	phenolics	eye, skin, and respiratory tract irritant. liver, kidney, and CNS toxicant
13	4-methyl-phenol	C_7_H_8_O	108.0575	4.744	96.7	798	phenolics	eye, skin, and respiratory tract irritant
14	2,4-dichlorophenol	C_6_H_4_Cl_2_O	161.9636	5.432	97.7	924	phenolics	liver and kidney toxicant

a RT: retention time; SI: NIST similarity/search index. Annotation score represents the overall compound identification confidence based on spectral similarity (SI), accurate mass agreement, and high-resolution filtering (HRF) criteria. (See Tables S2 and S3 for a detailed list of toxicants).

Phenolic compounds, including phenol, methylphenol, dimethylphenol, and chlorinated phenols, detected are key combustion-derived markers linked to lignin pyrolysis during biomass and wood burning.^27^ Their detection on wildland firefighter protective gear demonstrates the persistence of these semivolatile compounds within textile matrices. Tentatively identified agrochemical-associated compounds, including herbicides and pesticide-related compounds such as oxabetrinil, terbucarb, carbaryl, and *N*,*N*-diethyl-3-methylbenzamide (DEET), were also detected on firefighter protective pants based on spectral library matching in the absence of authentic analytical standards. These compounds likely represent environmentally associated contaminants rather than primary combustion markers, as firefighters may encounter treated vegetation, agricultural soils, insect repellents, and other environmental residues during prescribed burn operations. Although aliphatic hydrocarbons were abundant, this study primarily emphasized PAHs, alkylated PAHs, phenolic compounds, and aromatic hydrocarbons because of their greater toxicological significance and relevance to firefighter occupational exposure. Collectively, the detection of these chemical profiles agrees with field investigations showing that protective gear accumulates combustion products during fire response and in postfire off-gassing emissions.^16^ Similar chemical patterns were also observed for the colored firefighter pants (Figure S1b), indicating generally consistent contaminant profiles across both fabric types.

### 3.2 Effectiveness of Laundering as a Decontamination Strategy

PCA, boxplot, and hierarchical clustering analyses showed that laundered pants clustered closer to new pants, indicating that laundering effectively decontaminated each pair of firefighter gear (Figures 3, 4a, and S1a). The results revealed significant differences between dirty pants and washed pants (*p* < 0.001), as illustrated using toluene and the PAH compounds naphthalene, 1-methylnaphthalene, 2-methylnaphthalene, acenaphthene, and biphenyl (Figure 5). Selected contaminants were semiquantified using deuterated internal standards, and concentrations were expressed on a fabric-mass basis (ng/g). This approach is appropriate for comparing relative accumulation and removal across NP, DP, and WP samples. Comparisons between paired dirty-pants (DP) and washed-pants (WP) samples were evaluated using paired Student’s *t* tests, whereas comparisons between new fabric patch controls (NP) and exposed dirty-pants (DP) samples were evaluated using unpaired Student’s *t*-tests. For the selected compounds, noncolored firefighter pants revealed measurable accumulation of combustion-derived contaminants onto firefighter textiles during wildfire suppression activities following a single prescribed fire exposure event. Mean concentrations (±standard deviation, SD) of target compounds were substantially elevated in dirty pants relative to washed pants and new pants. Toluene and naphthalene concentrations in noncolored firefighter pants averaged 0.958 ± 0.298 ng/g and 0.053 ± 0.020 ng/g in dirty pants, respectively, while laundering substantially reduced concentrations in washed samples (Figure 5). A higher accumulation was observed for 1-methylnaphthalene, which showed mean concentrations of 0.468 ± 0.132 ng/g in dirty firefighter pants. Also, 2-methylnaphthalene was detected at a mean concentration of 0009 ± 0.002 ng/g in dirty pants. Lower concentrations of acenaphthene and biphenyl were observed. Acenaphthene concentrations averaged 0.003 ± 0.001 ng/g in dirty noncolored firefighter pants, while biphenyl concentrations averaged 0.015 ± 0.004 ng/g (Figure 5). The elevated concentrations observed in dirty pants relative to washed and new pants indicate substantial deposition and retention of combustion-derived aromatic compounds within firefighter textiles following wildfire exposure. Comparatively, colored firefighter pants exhibited higher contaminant concentrations than noncolored pants (Figure S2), suggesting greater retention of combustion-derived compounds within colored textile matrices.

**Figure 5:**
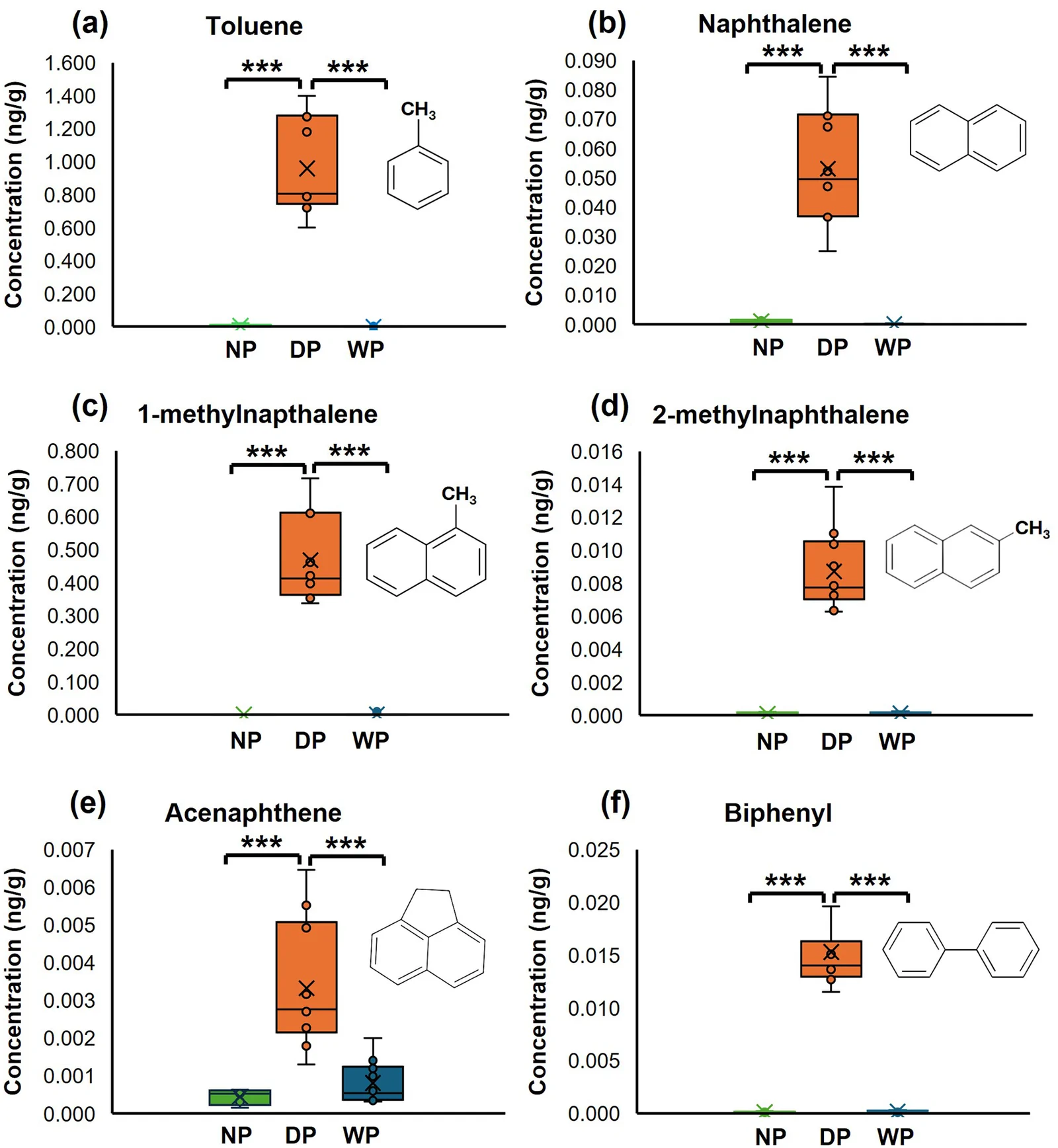
Semiquantitative concentrations of (a) toluene, (b) naphthalene, (c) 1-methylnaphthalene, (d) 2-methylnaphthalene, (e) acenaphthene, and (f) biphenyl detected in noncolored firefighter gear. DP: dirty pants; NP: new pants; WP: washed pants. (***P < 0.001).

Laundering after a single prescribed fire exposure event substantially reduced contaminant levels on firefighter protective pants, with washed pants (WP) consistently showing lower concentrations than dirty pants (DP) (Figure 5). Removal efficiency was calculated using paired dirty- and washed-pants concentrations according to [(DP – WP)/DP] × 100. Toluene exhibited very high laundering efficiency, with removal percentages ranging from 99.98% to 99.99%. Naphthalene, 1-methylnaphthalene, and 2-methylnaphthalene also showed high removal efficiency ranging from approximately 97.24% to 99.99%. Similarly, biphenyl demonstrated high laundering efficiency, with removal percentages ranging from 98.03% to 99.47%. In contrast, acenaphthene exhibited comparatively lower and more variable removal efficiencies ranging from 12.10% to 92.41% (Figure 6). Comparable trends were observed for colored firefighter pants, where lower molecular weight aromatic compounds generally exhibited removal efficiencies greater than 98% (i.e., 98.50% to 99.99%). Acenaphthene and biphenyl showed removal efficiencies ranging from 84.25% to 99.99% and 98.31% to 99.99%, respectively, indicating that laundering was highly effective at reducing many volatile and semivolatile combustion-derived contaminants following a single wildfire exposure event.

**Figure 6:**
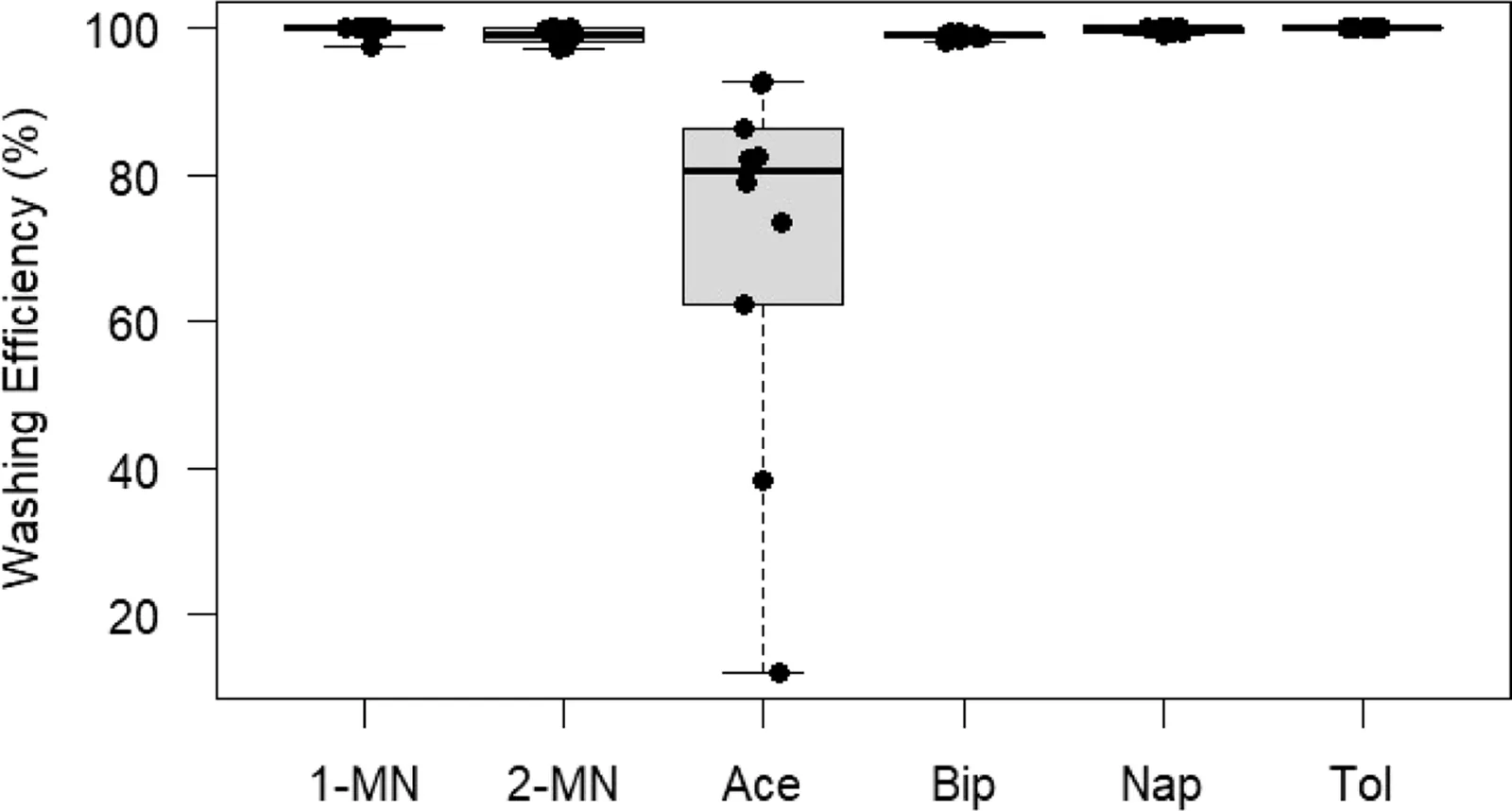
Washing efficiency (%) of selected combustion-derived contaminants detected in noncolored firefighter protective pants following a single prescribed fire exposure event and a single laundering cycle. 1-MN: 1-methylnaphthalene; 2-MN: 2-methylnaphthalene; Ace: acenaphthene; Bip: biphenyl; Nap: naphthalene; and Tol: toluene.

Despite the effectiveness of laundering to significantly reduce overall contamination, it fails to completely eliminate the contaminants on firefighter gear. This is evident from the residual contamination detectable after washing (Figure 5), particularly for heavier and more hydrophobic compounds such as acenaphthene and biphenyl. This suggests that certain aromatic contaminants may become more strongly sorbed within firefighter textile matrices and less efficiently removed during laundering. The persistence of residual contaminants after washing is consistent with previous firefighter exposure studies reporting incomplete removal of PAHs and combustion-derived toxicants from firefighter protective clothing, potentially contributing to prolonged secondary exposure through dermal contact, particle transfer, and off-gassing from contaminated gear.^10,28^ These findings emphasize the value of analyzing protective gear to capture the full range of exposure to firefighter toxicants through inhalation and dermal contact.

### 3.3 Health Implications of (Secondary) Exposure and Recommendations

While the textile-based concentrations (ng/g) measured in this study cannot be directly compared with airborne occupational exposure limits or toxicological guidance values reported in ppm or mg/m^3^, the detection and persistence of these combustion-derived compounds on firefighter textiles remain toxicologically relevant. Repeated handling of contaminated gear, dermal contact, particle transfer, and potential off-gassing may contribute to cumulative secondary exposure over time. For example, the Occupational Safety and Health Administration (OSHA) and National Institute for Occupational Safety and Health (NIOSH) have established occupational exposure limits for several compounds detected in firefighter textiles, including naphthalene, biphenyl, and toluene. OSHA and NIOSH report 8–10 h occupational exposure limits of 10 ppm (50 mg/m^3^) for naphthalene, while NIOSH recommends a short-term exposure limit of 15 ppm (75 mg/m^3^). The Agency for Toxic Substances and Disease Registry (ATSDR) has reported acute and intermediate-duration inhalation minimal risk levels (MRLs) for naphthalene and methylnaphthalene derivatives at substantially lower concentrations. Similarly, OSHA and NIOSH list occupational exposure limits of 0.2 ppm (1 mg/m^3^) for biphenyl and 200 and 100 ppm for toluene, respectively. Although these limits are based on airborne exposure scenarios rather than textile contamination, their existence highlights the recognized toxicological significance of compounds detected on firefighter protective gear.

Our findings revealed that wildland firefighter gear accumulated several known carcinogens and irritants. Specific volatile and semivolatile compounds of carcinogenic or suspected carcinogenic concern that were found on firefighter gear include naphthalene and alkylated naphthalene (Table 1). Since noncolored and colored pants were exposed to similar fire conditions and fuel composition and laundered using the same detergent, washing machine and dryer settings, cycle duration, and water temperature, the higher residual contaminant concentrations observed in some washed colored pants compared with noncolored pants may reflect variability in fire exposure conditions, incomplete removal of strongly retained compounds, contaminant redistribution during laundering, and possible contributions from dye- or detergent-related residues following washing. In addition, laundering may redistribute contaminants within textile fibers, which may create spatially heterogeneous postwash chemical profiles despite an overall reduction in contaminant levels.^29^ These findings indicate the need for periodic replacement or improved decontamination of firefighter gear. The persistence of residual contaminants on firefighter gear is particularly concerning, given the repeated and prolonged exposure after fire suppression. The detection of these compounds on firefighter textiles underscores the importance of secondary exposure risks associated with contaminated protective gear.

Toluene is classified as a skin irritant and reproductive toxicant. Acute inhalation exposure to toluene may cause respiratory irritation and central nervous system depression. Although toluene is not classified as a human carcinogen, prolonged or repeated exposure has been associated with neurotoxicity, dermatitis, and potential liver and kidney damage.^30,31^ Naphthalene is classified by the International Agency for Research on Cancer (IARC) as a Group 2B carcinogen (possibly carcinogenic to humans).^32^ Acute exposure of humans to naphthalene by inhalation and dermal contact is associated with skin and respiratory irritation, hemolytic anemia, damage to the liver, and, in infants, neurological damage, while chronic exposure is associated with laryngeal and intestinal cancer development.^33,34^ Other effects of chronic exposure to naphthalene include cataracts, lung inflammation, respiratory metaplasia, and retinal hemorrhage.^35^ Alkyl-naphthalene, such as 1-methylnaphthalene and 2-methylnaphthalene, often co-occurs with naphthalene in combustion mixtures, and their inhalation can cause respiratory and neurological effects in humans, similar to those seen with naphthalene, though the specific health effects of 1-methylnaphthalene and 2-methylnaphthalene are less well-studied.^36,37^

Several higher molecular weight aromatic compounds and PAHs found in this study, such as acenaphthene, biphenyl, acenaphthylene, fluorene, and phenanthrene are also known to cause skin, eye, and respiratory tract irritation, with some linked to neurological and central nervous system effects at high exposure levels (Table 1). Exposure to acenaphthene may irritate the respiratory tract, while repeated inhalation exposure has been associated with bronchitis characterized by persistent coughing and shortness of breath. Although acenaphthene is not currently classified as a human carcinogen, exposure has been associated with DNA methylation changes considered potential biomarkers of carcinogenic and systemic toxicological effects.^38^ Biphenyl (also known as diphenyl), as an aromatic hydrocarbon, is associated with skin, eye, and respiratory tract irritation following inhalation or dermal exposure. Repeated exposure has been linked to dermatitis, bronchitis, and neurological symptoms, and central nervous system effects, while prolonged exposure may also contribute to liver and kidney toxicity.^39^

While fluorene is not classified as a human carcinogen (group 3) by IARC, a study involving chimney sweeps exposed to PAHs, including fluorene, linked fluorene exposure with DNA methylation changes, potential markers for lung cancer.^40^ Fluorene is recognized as respiratory and skin irritants.^41,42^ Exposure to acenaphthylene, a PAH commonly found in fire smoke, can cause acute health effects such as irritation to the skin, eyes, and nose. Repeated and prolonged exposure can lead to respiratory issues, such as bronchitis, characterized by a persistent cough, phlegm, and shortness of breath, indicating a respiratory health burden among firefighters.^43^ Phenolic compounds detected, including phenol, alkylated phenols, and chlorinated phenols, are associated with significant irritant and systemic toxicological effects following inhalation or dermal exposure. Phenol exposure may cause severe skin and eye irritation or burns, while prolonged exposure has been associated with liver, kidney, and nervous system toxicity.^44^ Similarly, 3-methylphenol (*m*-cresol) and 4-methylphenol (*p*-cresol) are corrosive phenolic compounds capable of causing skin and respiratory irritation, pulmonary effects, and central nervous system symptoms following repeated exposure. Repeated skin contact with cresols may also contribute to dermatitis and systemic toxicity due to rapid dermal absorption. Chlorinated phenols, including 2,4-dichlorophenol and 4-chloro-3-methylphenol (chlorocresol), are strong skin, eye, and respiratory irritants and have been associated with mucosal inflammation, central nervous system depression, and potential liver and kidney toxicity following prolonged exposure.^45^ Their presence on firefighter textiles may therefore contribute to secondary exposure through repeated handling, dermal contact, and off-gassing from contaminated pants.

Studies of other organic pollutants have shown that of the three major routes of exposure, namely inhalation, ingestion, and dermal contact, inhalation constitutes a significant exposure route to airborne toxicants.^46^ Considering that firefighters are in regular contact with volatile and semivolatile toxicants, this study emphasizes the need for the consistent and proper use of personal protective equipment suitable for wildland firefighting conditions. While a self-contained breathing apparatus (SCBA) provides a continuous supply of fresh, breathable air in structural firefighting environments, its routine use during wildland fire operations is often infeasible due to remote locations, prolonged shifts, elevated temperatures, and the physical burden associated with additional equipment. At present, no standardized respiratory protection exists for wildland firefighters during wildland fires. Although some may use bandanas and/or face masks,^24^ many go unprotected during these events. This highlights a critical gap in occupational health protection and underscores the need for further research and policy development focused on practical, lightweight, and operationally feasible respiratory protection strategies for wildland firefighters.

Third-hand smoke has been demonstrated to persist on fabrics and off-gas long after initial exposures.^47^ Hence, wildland firefighter protective gear should not be worn in transport vehicles or living spaces, since exposure is prolonged by these behaviors. Current practices in wildland firefighting often involve sustained use of smoke-exposed gear over multiple days due to long deployments in rugged conditions. When possible, wearing laundered gear is still preferable to unlaundered smoke-exposed gear. Additionally, while unexamined at present, sleeping in spaces that also contain gear may present additional risks. When possible, wildland firefighting pants should be stored in separate spaces from sleeping quarters.

In view of the compounds detected, our results underscore the need for safe transport of these protective materials (e.g., in sealed bags), their proper storage prior to laundering, and highlight the importance of laundering firefighter gear immediately after occupational activities and separate from other materials. Since firefighter gear serves as a reservoir of occupational toxicants even after laundering, our results highlight the need for periodic replacement of gear even in the absence of visible wear and tear to limit secondary exposure to firefighters. While implementation of these practices may be challenging during prolonged deployments or in remote firefighting environments with limited resources, they remain important exposure-reduction strategies and warrant consideration in operational planning and postincident protocols. These practices may help reduce contaminant persistence and potential occupational exposure associated with firefighter protective gear.

## 4 Study Limitations

One limitation of the present study is that contaminant accumulation and laundering effectiveness were evaluated using attached firefighter fabric patches following a single prescribed burn exposure event and a single laundering cycle. Therefore, the observed contaminant profiles may not fully represent contamination patterns across entire firefighter garments or under different wildfire conditions, fuel compositions, combustion intensities, climatic conditions, laundering practices, and firefighter protective textile materials used across operational settings. In addition, fireground exposure conditions are inherently heterogeneous, and contaminant deposition on firefighter textiles may vary depending on smoke exposure intensity, fire behavior, operational activities, and duration of exposure during prescribed burning. Another limitation of this study is that textile swatches (including NP, DP, and WP) were transported in plastic bags. While this may result in the adsorption or loss of some volatile organic compounds (VOCs) and semivolatile organic compounds (SVOCs), any potential effect would be systematic rather than biasing one treatment group over another. Although all samples were handled consistently, future studies will use precombusted aluminum foil or precleaned glass containers for sample storage so as to minimize potential analyte loss during transport. In addition, GC–MS data were acquired over the *m*/*z* 50–500 range. Consequently, compounds producing ions above *m*/*z* 500 may not have been detected, potentially limiting the characterization of higher-mass SVOCs.

A broad range of volatile and semivolatile combustion-derived contaminants were putatively and tentatively identified using nontarget GC-EI Orbitrap analysis. However, only selected toxic compounds were semiquantified using deuterated internal standards. Future studies incorporating targeted quantitative analyses and authentic reference standards would further strengthen compound confirmation and exposure characterization. Furthermore, the present study did not directly evaluate inward contaminant penetration, dermal absorption, inhalation exposure from revolatilized compounds, or biologically relevant internal dose associated with contaminated firefighter textiles. Therefore, the findings should be interpreted as evidence of contaminant presence, persistence, and relative removal following laundering rather than direct measures of internal exposure or health risk. Future studies incorporating repeated exposure cycles, different fuel types, additional laundering conditions, and biological exposure assessment are warranted to further characterize occupational exposure pathways associated with wildland firefighter protective gear.

## Supplementary Material



## Notes

Summary of GC-EI orbitrap deconvolution, peak detection, grouping, and compound annotation parameters (Table S1); chemical profile of prescribed fire for colored pants (Figure S1); putatively identified volatile and semivolatile organic compounds detected in noncolored and colored firefighter pants (Table S2 and S3); and semiquantitative concentrations of selected toxicants in the colored firefighter gear (Figure S2) (PDF)

## References

[1] Ryan K. C., Knapp E. E., Varner J. M. (2013). Prescribed fire in North American
forests and woodlands: history, current practice, and challenges. Front. Ecol. Environ..

[2] Robinson M. (2008). Occupational PAH exposures during prescribed pile burns. Ann. Occup. Hyg..

[3] Patel A. B. (2020). Polycyclic aromatic hydrocarbons: sources, toxicity, and remediation
approaches. Front. Microbiol..

[4] Guo Y. (2011). International perspectives: sources, distribution,
and toxicity of
polycyclic aromatic hydrocarbons. J. Environ.
Health.

[5] Wu C.-M. (2021). Characterization
of occupational smoke exposure among wildland firefighters
in the midwestern United States. Environ. Res..

[6] Navarro K. M. (2018). A review of community smoke exposure from wildfire
compared to prescribed
fire in the United States. Atmosphere.

[7] Robinson M. S. (2011). Characterization of PM2.5 collected during broadcast and slash-pile
prescribed burns of predominately ponderosa pine forests in northern
Arizona. Atmos. Environ..

[8] Hu Y. (2008). Simulation of Air Quality
Impacts from Prescribed Fires on an Urban
Area. Environ. Sci. Technol..

[9] Navarro K. M. (2016). Air-Quality Impacts and Intake Fraction of PM2.5 during the 2013
Rim Megafire. Environ. Sci. Technol..

[10] Fent K. W. (2017). Contamination of firefighter
personal protective equipment and skin
and the effectiveness of decontamination procedures. J. Occup. Environ. Hyg..

[11] Baxter C. S. (2014). Exposure of firefighters to particulates and
polycyclic aromatic
hydrocarbons. J. Occup. Environ. Hyg..

[12] Aliaño-González M. J. (2022). Assessment of volatile compound transference through firefighter
turnout gear. Int. J. Environ. Res. Public Health.

[13] Lee D. J., Ahn S., McClure L. A., Caban-Martinez A. J., Kobetz E. N., Ukani H., Boga D. J., Hernandez D., Pinheiro P. S. (2023). Cancer risk and
mortality among firefighters: a meta-analytic review. Front. Oncol..

[14] Demers P. A. (2022). Carcinogenicity of occupational exposure as a firefighter. Lancet Oncol..

[15] Banks A. P. W. (2021). Assessing decontamination and laundering processes
for the removal of polycyclic aromatic hydrocarbons and flame retardants
from firefighting uniforms. Environ. Res..

[16] Wilkinson A. F., Fent K. W., Mayer A. C., Chen I. C., Kesler R. M., Kerber S., Smith D. L., Horn G. P. (2023). Use of
Preliminary
Exposure Reduction Practices or Laundering to Mitigate Polycyclic
Aromatic Hydrocarbon Contamination on Firefighter Personal Protective
Equipment Ensembles. Int. J. Environ. Res. Public
Health.

[17] Li H. (2022). Non-targeted analysis
of unknown volatile components in scented stationery
via headspace GC-Orbitrap MS. Talanta.

[18] Kwiecien N. W. (2015). High-Resolution Filtering for Improved Small
Molecule Identification
via GC/MS. Anal. Chem..

[19] Schymanski E. L. (2014). Identifying Small Molecules
via High Resolution Mass Spectrometry:
Communicating Confidence. Environ. Sci. Technol..

[20] Austin C. C. (2001). Characterization of
volatile organic compounds in smoke at municipal
structural fires. Journal of Toxicology and
Environmental Health Part A.

[21] Navarro K. M. (2017). Occupational Exposure to Polycyclic Aromatic
Hydrocarbon of Wildland
Firefighters at Prescribed and Wildland Fires. Environ. Sci. Technol..

[22] Navarro K. (2020). Working in
Smoke:: Wildfire Impacts on the Health of Firefighters and Outdoor
Workers and Mitigation Strategies. Clin Chest
Med..

[23] Teixeira J., Sousa G., Azevedo R., Almeida A., Delerue-Matos C., Wang X., Santos-Silva A., Rodrigues F., Oliveira M. (2024). Characterization of Wildland Firefighters’
Exposure
to Coarse, Fine, and Ultrafine Particles; Polycyclic Aromatic Hydrocarbons;
and Metal­(loid)­s, and Estimation of Associated Health Risks. Toxics.

[24] Hwang J., Chong N. S., Zhang M., Agnew R. J., Xu C., Li Z., Xu X. (2023). Face-to-face
with scorching wildfire: potential toxicant
exposure and the health risks of smoke for wildland firefighters at
the wildland-urban interface. The Lancet Regional
Health.

[25] Sousa G., Teixeira J., Delerue-Matos C., Sarmento B., Morais S., Wang X., Rodrigues F., Oliveira M. (2022). Exposure to PAHs during
Firefighting Activities: A Review on Skin Levels, In Vitro/In Vivo
Bioavailability, and Health Risks. Int. J. Environ.
Res. Public Health.

[26] Mayer A. C. (2022). Characterizing exposure
to benzene, toluene, and naphthalene in firefighters
wearing different types of new or laundered PPE. Int. J. Hyg. Environ. Health.

[27] Hartikainen A. (2020). Photochemical transformation
of residential wood combustion emissions:
dependence of organic aerosol composition on OH exposure. Atmos. Chem. Phys..

[28] Mayer A. C. (2019). Firefighter hood contamination: Efficiency
of laundering to remove
PAHs and FRs. J. Occup. Environ. Hyg..

[29] Horn G. P. (2021). Impact of repeated exposure
and cleaning on protective properties
of structural firefighting turnout gear. Fire
Technol..

[30] Occupational Safety and Health Administration (OSHA) . Toluene: Occupational Exposure Limits, 2022. Available from: https://www.osha.gov/toluene/occupational-exposure-limits. (accessed October 7, 2025).

[31] NIOSH . NIOSH Pocket Guide to Chemical Hazards: Toluene, 2024. Available from: https://www.cdc.gov/niosh/npg/npgd0619.html. 11326577

[32] IARC . Overall Evaluations of Carcinogenicity An Updating of IARC Monographs; International Agency for Research on Cancer: Lyon, France, 1987. (accessed October 7, 2025).

[33] IARC Working Group on the Evaluation of Carcinogenic Risks to Humans and World Health Organization and International Agency for Research on Cancer . Some Traditional Herbal Medicines. Some Mycotoxins, Naphthalene and Styrene; World Health Organization, 2002; Vol, 82, pp 1–556. PMC4781602 12687954

[34] Yost E. E., Galizia A., Kapraun D. F., Persad A. S., Vulimiri S. V., Angrish M., Lee J. S., Druwe I. L. (2021). Health Effects of
Naphthalene Exposure: A Systematic Evidence Map and Analysis of Potential
Considerations for Dose-Response Evaluation. Environ. Health Perspect..

[35] USEPA . Health and Environmental Effects Profile for Naphthalene. EPA/600/x-86/241; Environmental Criteria and Assessment Office, Office of Health and Environmental Assessment, Office of Research and Development: Cincinnati, OH, 1986.

[36] Lin C. Y. (2009). Toxicity and metabolism of methylnaphthalenes: comparison with naphthalene
and 1-nitronaphthalene. Toxicology.

[37] ATSDR . Toxicological Profile for Naphthalene (Update); Public Health Service, U.S. Department of Health and Human Services: Atlanta, GA, 1995.

[38] NJDOH . Hazardous Substance Fact Sheet: Acenaphthene; Department of Health and Senior Services: NJ, 2007.

[39] EPA, USEPA . Biphenyl Hazard Summary; United States Environmental Protection Agency (EPA), 2000.

[40] Alhamdow A. (2020). Fluorene exposure among
PAH-exposed workers is associated with epigenetic
markers related to lung cancer. Occup. Environ.
Med..

[41] NJDHSS . Hazardous Substance Fact Sheet: Fluorene; Department of Health and Senior Services: Trenton, NJ, 1999.

[42] ECHA . 9,9-Dimethyl-9H-fluorene (EC: 893–658–0); European Chemicals Agency: Helsinki, Finland, 2025.

[43] Teixeira J. (2024). Firefighters’ personal exposure to gaseous PAHs during controlled
forest fires: A case study with estimation of respiratory health risks
and in vitro toxicity. Sci. Total Environ..

[44] NJDOH . Hazardous Substance Fact Sheet: P-Chloro-M-Cresol; Department of Health and Senior Services: NJ, 2007.

[45] NJDOH . Hazardous Substance Fact Sheet: Cresols (Mixed Isomers); Department of Health and Senior Services: NJ, 2007.

[46] Saliu T. D. (2025). Occurrence of PFAS in Lyon soils: contribution
and impact of industrial
activities. Environ. Sci.:Adv..

[47] Borujeni E. T. (2022). Identification and determination of the volatile organics of third-hand
smoke from different cigarettes and clothing fabrics. J. Environ. Health Sci. Eng..

