# Supplementary material for: Nontarget Analysis of the Volatile and Semivolatile Chemical Profile of Wildland Firefighter Gear: Insight into Occupational Exposure and Health Implications
Source: Environ Sci Technol. 2026 Aug 21;60(38):27383–94. doi: 10.1021/acs.est.6c10862 (PMC13629623; doi:10.1021/acs.est.6c10862)
Supplement: Supplementary file 1 [file es6c10862_si_001.pdf]

Non-target analysis of the volatile and semi-volatile chemical profile of wildland firefighter gear: Insight into occupational exposure and health implications

Patrica-Ivy Agorsor[1], Husam Kafeenah[1], Margaret D. Taiwo[1], Robin Verble[2,3] and Michael O. Eze[1,3,4*]

[1]Department of Chemistry, Missouri University of Science and Technology, Rolla, MO 65409, USA.
[2]Department of Biological Sciences, Missouri University of Science and Technology, Rolla, MO 65409, USA.
[3]Center for Research in Energy and Environment, Missouri University of Science and Technology, Rolla, MO 65409, USA.
[4]Center for Biomedical Research, Missouri University of Science and Technology, Rolla, MO 65409, USA.

*Correspondence: meze@mst.edu

Supplementary Information

Table S1. Summary of GC-EI Orbitrap deconvolution, peak detection, grouping, and compound annotation parameters used in Compound Discoverer™ for non-target analysis of volatile and semi-volatile organic compounds in firefighter protective pants.

Category	Key Parameters	Settings
Peak Detection	Mass tolerance; spectral S/N threshold; peak S/N threshold; smoothing factor; TIC threshold; ion overlap window	5 ppm; 3; 3; 9; 1×10^6; 99
Retention Index (RI)	RI calculation; column type	Enabled; semi-non-polar column
Compound Grouping	Retention Time (RT) tolerance; composition threshold	5 s; 10
Library Search	Search type; matching threshold, reverse search; high-resolution filtering (HRF)	HiRes; SI/RSI $\geq$ 700; enabled; enabled
Filtering & Annotation	Fragment matching; missing ion penalty; synonym search; RI filtering	Enabled

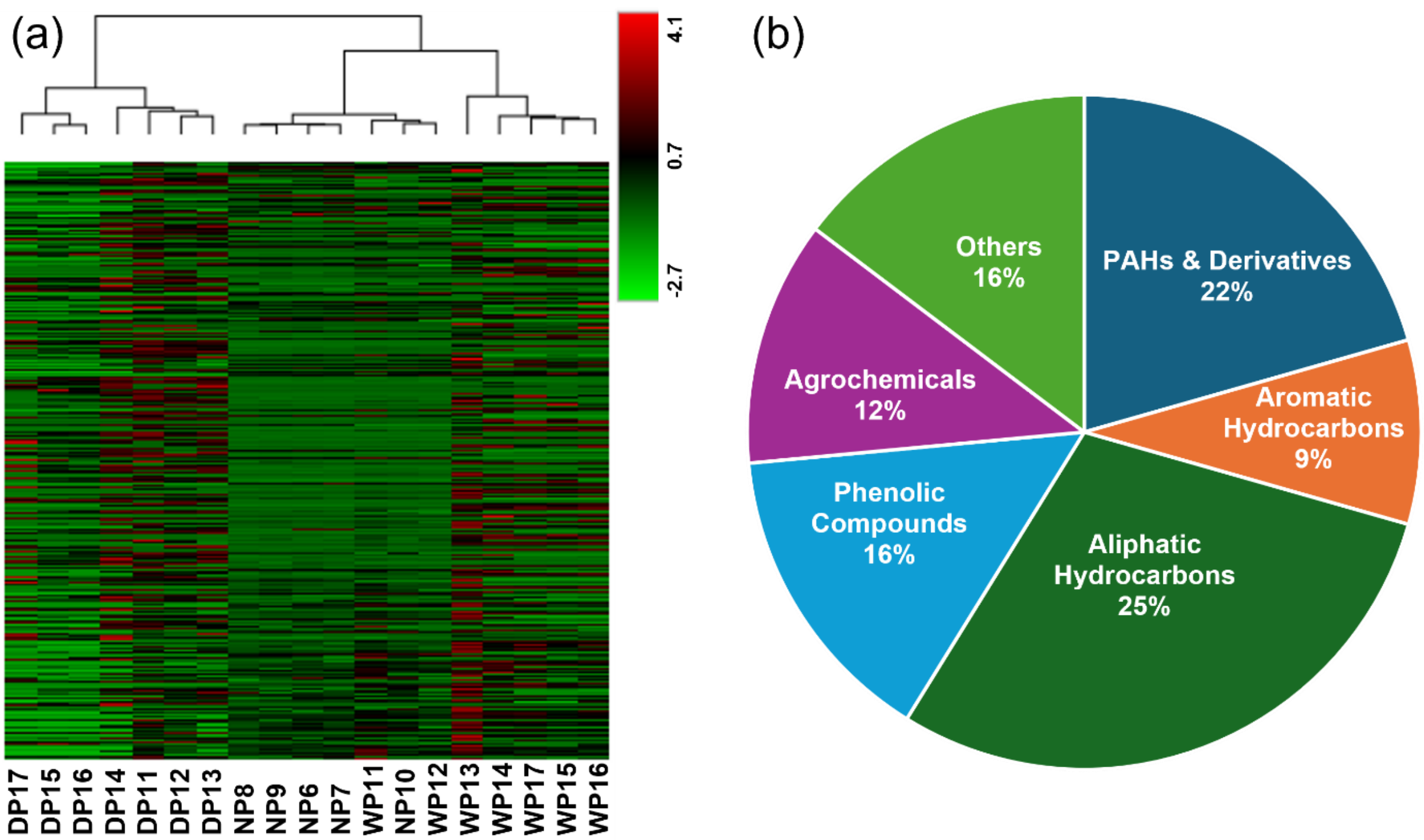

Fig. S1. Chemical profile of prescribed fire. (a) Heat map showing hierarchical clustering of new, washed, and dirty colored pants. The color scale indicates relative chemical abundance, with red signifying high chemical intensity. (b) Percentage distribution of the dominant chemical class in the colored firefighter pants. NP: New pants; WP: Washed pants; DP: Dirty pants.

Table S2. Putatively identified volatile and semi-volatile organic compounds detected in non-colored firefighter pants.

Compounds	Molecular Weight	RT (min)	Formula	Annotation Score	SI	HRF Score	ΔRI
**Aromatic Hydrocarbons**							
Toluene	92.0618	2.724	C_7H_8	76.5	808	99.7	
(1,1-Dimethylpropyl)benzene	148.1247	5.285	$C_{11}H_{16}$	71.2	709	88.6	24
Pentylbenzene	148.1248	5.536	$C_{11}H_{16}$	77.4	849	99.8	23
**PAHs & Derivatives**							
Naphthalene	128.0624	5.581	$C_{10}H_8$	96.8	932	95.3	8
1-Methylnaphthalene	142.0783	6.244	$C_{11}H_{10}$	99.9	957	97.6	78
2-Methylnaphthalene	142.0784	6.338	$C_{11}H_{10}$	98.7	960	96.8	53
Biphenyl	154.0780	6.699	$C_{12}H_{10}$	98.4	935	96.7	85
Acenaphthylene	152.0625	7.968	$C_{12}H_8$	92.8	702	84.7	28
Acenaphthene	154.0781	8.590	$C_{12}H_{10}$	98.8	722	93.9	8
Fluorene	166.0783	9.126	$C_{13}H_{10}$	91.2	729	81.9	32
Phenanthrene	178.0791	10.251	$C_{14}H_{10}$	84.5	768	79.5	88
**Aliphatic Hydrocarbons**							
n-Decane	142.1716	4.359	$C_{10}H_{22}$	78.6	930	99.9	9
n-Undecane	156.1873	4.734	$C_{11}H_{24}$	78.2	911	99.2	33
n-Dodecane	170.2029	5.814	$C_{12}H_{26}$	78.8	920	98.0	19
n-Tridecane	184.2186	7.012	$C_{13}H_{28}$	75.7	797	92.8	41
n-Tetradecane	198.2342	7.114	$C_{14}H_{30}$	77.0	828	98.2	69
n-Pentadecane	212.2499	8.743	$C_{15}H_{32}$	76.3	825	95.6	18
n-Heptadecane	240.2812	9.408	$C_{17}H_{36}$	76.5	860	97.5	99
n-Octadecane	254.2968	9.509	$C_{18}H_{38}$	75.7	705	84.6	86
n-Eicosane	282.3281	10.308	$C_{20}H_{42}$	79.4	967	99.9	
**Phenolic Compounds**							
Phenol	94.0416	4.105	C_6H_6O	99.6	972	99.5	
4-Methylphenol	108.0575	4.744	C_7H_8O	96.7	798	97.8	1
3-Methylphenol	108.0570	5.180	C_7H_8O	95.6	830	89.4	2
2,4-Dimethylphenol	122.0727	5.238	$C_8H_{10}O$	97.5	880	98.4	2
2,4-Dichlorophenol	161.9636	5.432	$C_6H_4Cl_2O$	97.7	924	96.7	5
4-Chloro-3-methylphenol	142.0186	6.089	C_7H_7ClO	98.2	923	99.3	44
3-Hydroxybiphenyl	170.0731	7.408	$C_{12}H_{10}O$	96.0	727	83.0	
**Agrochemicals**							
DEET	191.1305	11.023	$C_{12}H_{17}NO$	79.2	972	99.1	17
Carbaryl	201.0784	11.973	$C_{12}H_{11}NO_2$	74.1	774	76.8	
Oxabetrinil	232.0842	13.308	$C_{12}H_{12}N_2O_3$	76.0	797	100	

Terbucarb	277.2036	15.160	$C_{17}H_{27}NO_2$	72.4	882	99.0	

HRF: High-resolution filtering; SI: NIST similarity/search index; RT: Retention time; ΔRI: Delta retention index; DEET: N,N-Diethyl-3-methylbenzamide

Table S3. Putatively identified volatile and semi-volatile organic compounds detected in colored firefighter pants.

Compounds	Molecular Weight	RT (min)	Formula	Annotation Score	SI	HRF Score	ΔRI
**Aromatic Hydrocarbons**							
(1,1-Dimethylpropyl)benzene	148.1247	5.285	$C_{11}H_{16}$	79.5	874	84.4	61
Pentylbenzene	148.1248	5.536	$C_{11}H_{16}$	76.2	753	89.4	
1,3-Di-tert-butylbenzene	190.1716	6.571	$C_{14}H_{22}$	94.6	736	87.0	30
**PAHs & Derivatives**							
Naphthalene	128.0624	5.581	$C_{10}H_8$	97.0	957	94.6	9
1-Methylnaphthalene	142.0783	6.244	$C_{11}H_{10}$	98.8	894	96.3	76
2-Methylnaphthalene	142.0784	6.338	$C_{11}H_{10}$	98.9	964	96.3	51
Acenaphthylene	152.0625	7.968	$C_{12}H_8$	95.4	855	87.9	13
Biphenyl	154.0780	6.699	$C_{12}H_{10}$	98.0	963	96.4	85
Acenaphthene	154.0781	8.590	$C_{12}H_{10}$	93.9	737	80.7	77
Fluorene	166.0783	9.126	$C_{13}H_{10}$	70.8	762	84.9	16
**Aliphatic Hydrocarbons**							
n-Decane	142.1716	4.359	$C_{10}H_{22}$	74.4	746	98.2	
n-Undecane	156.1873	4.734	$C_{11}H_{24}$	77.6	875	99.3	33
n-Dodecane	170.2029	5.814	$C_{12}H_{26}$	74.9	728	82.6	3
n-Tridecane	184.2186	7.012	$C_{13}H_{28}$	74.5	706	97.1	2
n-Tetradecane	198.2342	7.114	$C_{14}H_{30}$	74.4	751	96.0	28
n-Pentadecane	212.2499	8.743	$C_{15}H_{32}$	76.3	715	79.3	68
n-Octadecane	254.2968	9.509	$C_{18}H_{38}$	75.7	785	98.8	57
n-Eicosane	282.3281	10.308	$C_{20}H_{42}$	75.9	782	100	
**Phenolic Compounds**							
Phenol	94.0416	4.105	C_6H_6O	99.6	985	99.7	
4-Methylphenol	108.0575	4.744	C_7H_8O	95.4	792	97.7	1
3-Methylphenol	108.0570	5.180	C_7H_8O	99.3	965	100	24
2,4-Dimethylphenol	122.0727	5.238	$C_8H_{10}O$	96.4	784	91.2	1
2,6-Di-tert-butylphenol	206.1669	7.286	$C_{14}H_{22}O$	98.3	920	99.5	86
**Agrochemicals**							
DEET	191.1305	11.023	$C_{12}H_{17}NO$	71.3	816	99.5	
Carbaryl	201.0784	11.973	$C_{12}H_{11}NO_2$	71.1	791	100	

Oxabetrinil	232.0842	13.308	$C_{12}H_{12}N_2O_3$	75.5	785	99.2	
Terbucarb	277.2036	15.160	$C_{17}H_{27}NO_2$	78.2	717	100	
**Others**							
1,1'-Bicyclohexyl	166.1716	6.297	$C_{12}H_{22}$	73.7	713	95.4	69
Benzyl alcohol	108.0574	4.751	C_7H_8O	93.4	808	92.6	35
4-Chloroaniline	127.0193	5.642	C_6H_6ClN	98.5	844	83.2	2
1,4-Dichlorobenzene	145.9685	4.882	$C_6H_4Cl_2$	93.6	913	82.5	27
Bis(2-chloroethoxy)methane	172.0052	6.466	$C_5H_{10}Cl_2O_2$	78.7	882	96.2	22

HRF: High-resolution filtering score; SI: NIST similarity/search index; RT: Retention time; ΔRI: Delta retention index.

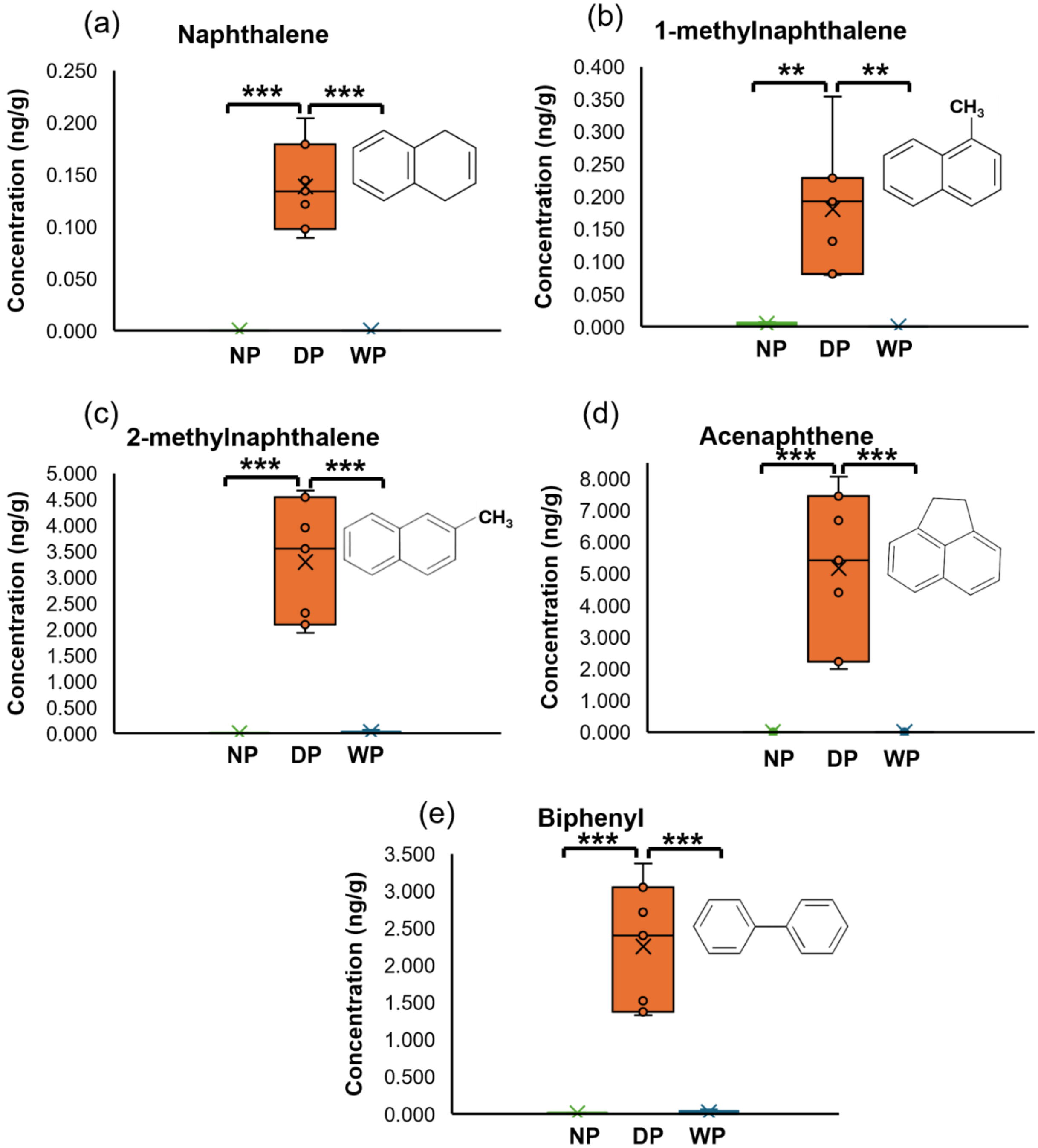

Fig. S2. Semi-quantitative concentrations of (a) naphthalene, (b) 1-methylnaphthalene, (c) 2-methylnaphthalene, (d) acenaphthene, and (e) biphenyl detected in the colored firefighter gear. DP: Dirty pants; NP: New pants; WP: Washed pants. (***P* < 0.01; ****P* < 0.001).
